# *egfl6* expression in the pharyngeal pouch is dispensable for craniofacial development

**DOI:** 10.1080/19768354.2021.1970018

**Published:** 2021-08-27

**Authors:** Sil Jin, Hyejee Na, Haewon Jeon, Jangwon Park, Chong Pyo Choe

**Affiliations:** a Division of Applied Life Science, Plant Molecular Biology and Biotechnology Research Center, Gyeongsang National University, Jinju, South Korea; b Division of Life Science, Gyeongsang National University, Jinju, South Korea

**Keywords:** *egfl6*, pharyngeal pouch, craniofacial development, zebrafish

## Abstract

Epidermal growth factor-like domain multiple 6 (Egfl6) is a basement membrane protein and plays an important role in hair follicle morphogenesis, angiogenesis, notochord development in vertebrates. Although *egfl6* expression in the developing head was observed in zebrafish, its role for craniofacial development and the determination of the pharyngeal region expressing *egfl6*, have not been reported yet. Here, we report the expression patterns and function of *egfl6* in craniofacial development in zebrafish. *egfl6* was expressed sequentially in the developing pharyngeal pouches that are key epithelial structures governing the development of the vertebrate head. However, loss-of-function mutations in *egfl6* did not cause any craniofacial defects, including the pouches as well as the thymus and facial cartilages whose development is contingent upon appropriate pouch formation. *egfl6* was unlikely redundant with *egfl7* expressed in a distinct pharyngeal region from that of *egfl6* in craniofacial development because reduction of *egfl7* with a MO in *egfl6* mutants did not affect craniofacial development. In addition, we found that *egfl6* carried an endogenous start loss mutation in the wild-type Tübingen strain, implying *egfl6* would be a non-functional gene. Taken all together, we suggest that *egfl6* expression in the pharyngeal pouches is not required for craniofacial development in zebrafish.

## Introduction

In vertebrates, a series of epithelial branches termed pharyngeal pouches arises in the pharyngeal endoderm during craniofacial development (Grevellec and Tucker 2010). Zebrafish forms six pouches, with mice and humans forming five, in the embryonic head (Graham 2008). The pouches segment the neural crest-derived pharyngeal arches sequentially, then provide signals, such as Sonic Hedgehog and Jagged, for the arch cells to be survived and differentiate into the facial skeletons (Miller et al. 2000; Zuniga et al. 2010). In addition, a population of pouch cells becomes the rudiments of endocrine glands, such as the thymus and parathyroid (Grevellec and Tucker 2010). Consistent with the essential role of pouches in organizing the head, abnormal development of the third and fourth pouches in human causes DiGeorge syndrome (DGS) with features, including facial anomalies, hypoplastic thymus with immune deficit, palatal anomalies, neonatal hypocalcemia, and heart defect (Driscoll et al. 1992).

Recent studies carried out in mice and zebrafish are shedding light on the developmental mechanisms underlying pouch formation. Loss-of-function mutations in *tbx1* gene in mice and zebrafish show defects almost identical to those of people with DGS, including the loss of or abnormal pouches, facial anomalies, hypoplastic thymus, and heart defects. Accordingly, *tbx1* was determined as the DGS gene (Lindsay et al. 2001; Piotrowski et al. 2003). Genetically, Tbx1 interacts with Fgf3 and Fgf8 for pouch formation in mice and zebrafish (Crump et al. 2004a; Herzog et al. 2004; Aggarwal et al. 2006; Choe and Crump 2014). Besides, transcription factors Pax1/9 (Peters et al. 1998; Liu et al. 2020), Foxi1/3 (Nissen et al. 2003; Solomon et al. 2003; Edlund et al. 2014; Jin S et al. 2018), and Nkx2.3 (Li et al. 2019) are required for pouch formation in mice and zebrafish. In addition to Fgf, signaling pathways, such as Wnt (Choe et al. 2013), ephrin/Eph (Choe and Crump 2015), Integrin (Crump et al. 2004b), and BMP (Lovely et al. 2016; Li et al. 2019) have been implicated in pouch development in zebrafish. Interestingly, a single-cell RNA sequencing performed in zebrafish embryos revealed previously unidentified genes expressed in 24 h-post-fertilization (hpf) cells of pharyngeal endoderm (PE) (Wagner et al. 2018). These included *keratin 8*, *keratin 18*, *EGF-like-domain, multiple 6* (*egfl6*), and *nanos1* (Wagner et al. 2018). In order to better understand the genetic mechanism underlying the development of pouches in zebrafish, here we analyze the potential role of *egfl6* in pouch formation.

Since the first identification of *EGFL6* in human fetal tissues (Yeung et al. 1999), orthologs of *EGFL6*, also called *MAM and EGF containing gene* (*MAEG*), have been identified in vertebrates, with biological functions being analyzed during embryonic development in mice and zebrafish. In mice, *Egfl6* has been shown as a molecular marker for dermatome, with immunohistochemistry showing the distribution of Egfl6 in the basement membrane of developing hair follicles, in which Egfl6 serves as an adhesive ligand for the α8β1 integrin (Buchner et al. 2000; Osada et al. 2005; Fujiwara et al. 2011). In zebrafish embryos, *egfl6* is expressed in the developing somites, with the expression being expanded to the whole trunk; immunohistochemistry and knockdown experiments show that Egfl6 expression accumulated in the notochord is required for the normal development of notochord (Wang et al. 2015). Egfl6 secreted from the somites is also involved in embryonic angiogenesis (Wang et al. 2016). While *egfl6* expression is also seen in the developing hindbrain, pharyngeal region, and fin epidermis (Wang et al. 2015), the roles of *egfl6* in the development of these tissues have not yet been analyzed. We find the pouch-specific expression of zebrafish *egfl6* during pouch morphogenesis and no defects in the pouches and their derivatives by loss of *egfl6*.

## Materials and methods

### Zebrafish lines

All zebrafish work was approved by Gyeongsang National University Institutional Animal Care and Use Committee. Zebrafish were raised and maintained by the Animal Protection Act (2017), Korea. *Tg(*∼*3.4her5:EGFP)* (Tallafuss and Bally-Cuif 2003) and *Tg(sox:EGFP)* (Carney et al. 2006) lines used in this study were published. To generate *egfl6* mutant lines with CRISPR/Cas9 system, 150 pg of *in vitro*-synthesized gRNA and 900 pg *in vitro*-transcribed mRNA encoding a nuclear-localized Cas9 were injected into one-cell stage wild-type Tübingen (TU) embryos. To identify carriers with germline transmission deletions in the *egfl6* gene, embryos were raised to adulthood and outbred to wild-type TU zebrafish. The carriers for *egfl6* mutant alleles were in-crossed, and the resulting embryos were used for *in situ* hybridization, immunochemistry, and alcian blue staining. For genotyping of *egfl6* mutant alleles, PCR amplicons produced by primers *egfl6*_F (5′-CAGCCATGCATACACAAA-3′) and *egfl6*_R (5′-CTGTCAGTATGGGCTGCT-3′) were digested with TaqI; while a wild-type allele had 208 and 252 bp, *egfl6* mutant alleles generated 452 bp (*egfl6^GNU12^*), 455bp (*egfl6^GNU13^*), and 468 bp (*egfl6^GNU14^*). *egfl6*-morpholino (MO) and *egfl7*-MO published previously (Parker et al. 2004; Wang et al. 2015) were obtained from Genetools, and 1 nl of a 300-μM solution was injected at the one-cell-stage.

### Staining

Fluorescent *in situ* hybridizations in conjunction with GFP immunohistochemistry (NC9589665, Torrey Pines Biolabs, 1:1000), Alcama/ZN8 immunohistochemistry (AB_531,904, Zebrafish International Resource Center, 1:400), and Alcian Blue staining were performed as described previously (Crump et al. 2004a; Zuniga et al. 2011; Lee et al. 2020; Chowdhury et al. 2021). Partial cDNA fragments of *egfl6*, *egfl7*, and *rag1* were amplified from mixed-stage embryos and cloned into the pGEM^®^-T easy vector (A1360, Promega). Antisense riboprobes were synthesized with T7 or SP6 RNA polymerase (111,750,25910, Roche Life Sciences) using digoxigenin (DIG)-labeled nucleotides (Roche) from sequence-verified plasmids. See Table 1 for primers.

**Table 1.** List of primers used to generate *in situ* probes.

Gene	Forward primer (5′ to 3′)	Reverse primer (5′ to 3′)
*egfl6*	TGG GAC AGC AGT AAA GGA	ATC TTC CAG CAG GAG CTT
*egfl7*	ATC ACC ATG TGC CAA AAC	AAT TGG TTC GCT CAG ACA
*rag1*	AGA TTC AGG AGG GAC TCG	ACG GGT CAG TGA CAA CAG

### Imaging

Fluorescent images were acquired on an Olympus FV1000MPE confocal microscope. Approximately 100-μm-thick z-stacks were captured with an Olympus UPLFLN 10X Objective lens and were assembled using Fluoview Advanced Software. Facial cartilages dissected manually were imaged on an Olympus BX50 upright microscope using mosaic V2.1 software.

## Results

### Expression of *egfl6* in pharyngeal pouch morphogenesis

**Figure 1.**
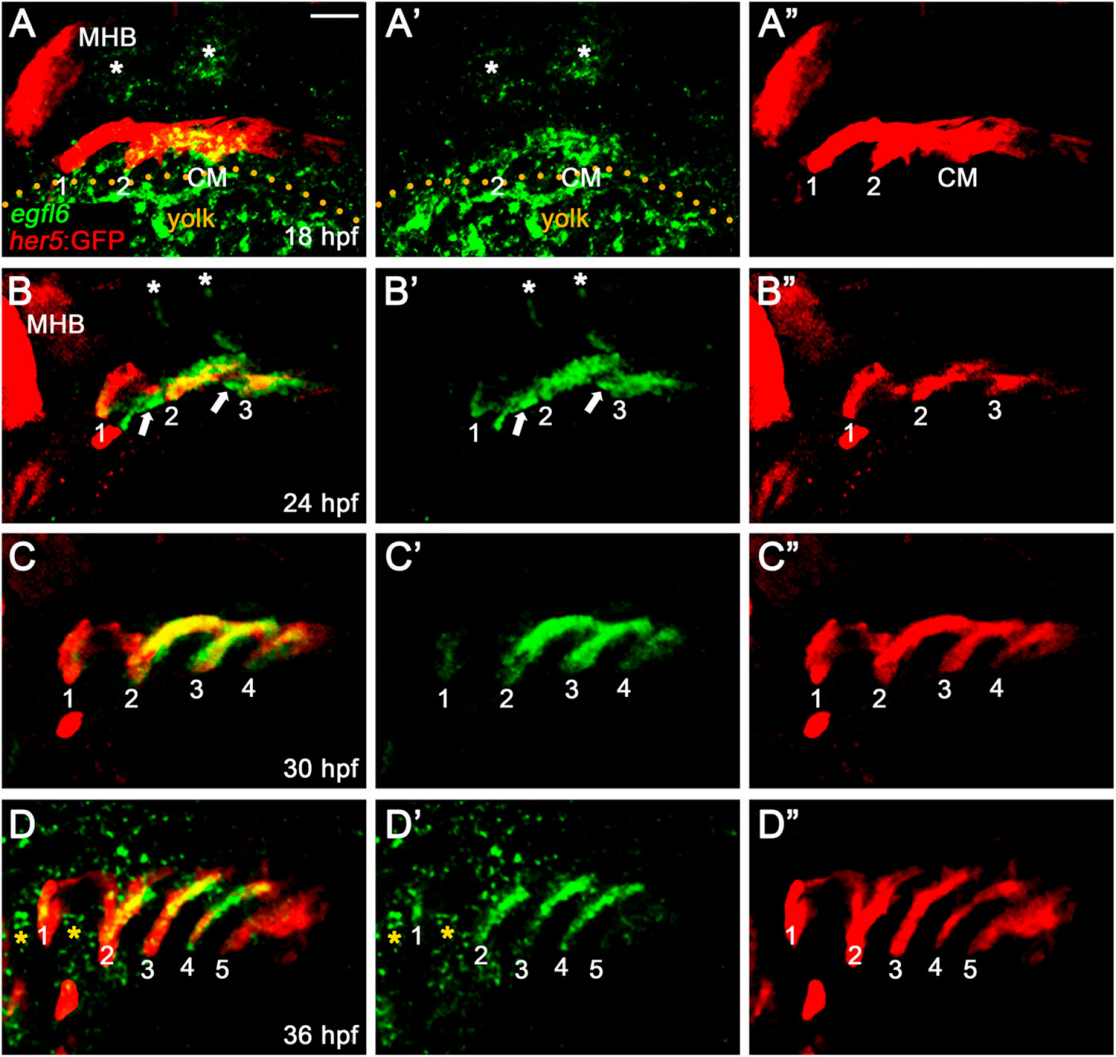
Expression of *egfl6* in pouch formation. (A-D) Fluorescence *in situ* hybridization of *egfl6* (green) in conjunction with the GFP immunohistochemistry (red) in wild-type *Tg*(*her5*:GFP) animals. (A) At 18 hpf, *egfl6* expression is seen in the *her5*-positive second (2) pouch and the posterior cell mass (CM), with no *egfl6* expression seen in the first (1) pouch. *egfl6* expression is also seen in the developing hindbrain (asterisks). Note of non-specific green staining in the yolks (dotted line). (B) At 24 hpf, *egfl6* is expressed in all three *her5*-positive pouches (1-3), with new *egfl6* expression appearing in the mesoderm between pouches (arrows). *egfl6* expression is still seen in the developing hindbrain (asterisks). (C) At 30 hpf, *egfl6* expression is only observed in all four *her5*-positive pouches (1-4), with the *egfl6* expression in the mesoderm gone. (D) At 36 hpf, *egfl6* is expressed in all pouches, with its expression in the fifth (5) pouch being faint. Also, unidentified tissues adjacent to the first (1) and second (2) pouches express *egfl6* (asterisks). Note that the sixth pouch is barely seen at the level of tissues. MHB: midbrain−hindbrain boundary. (A′–D′) Green channel only. (A”–D”) Red channel only. Scale bar: 40 μm.

To investigate the potential role of Egfl6 in pouch formation, we first analyzed the expression patterns of *egfl6* by *in situ* hybridization during pouch morphogenesis. In zebrafish, a total of six pouches form sequentially in the pharyngeal endoderm from 18 to 36 hpf, with the first two pouches forming simultaneously at 18 hpf and the sixth pouch hard to see at 36 hpf (Choe et al. 2013). We analyzed *egfl6* expression at 18, 24, 30, and 36 hpf in wild-type embryos harboring *Tg(her5:EGFP)* transgene that drives GFP expression in the pharyngeal endoderm and pouches (Tallafuss and Bally-Cuif 2003). At 18 hpf, *egfl6* was expressed in *her5*-positive pharyngeal endoderm, including the second pouch and posterior cell mass, with no *egfl6* expression being seen in the first pouch (Figure 1(A)). As previously reported (Wang et al. 2015), *egfl6* expression was observed in the developing hindbrain (asterisks in Figure 1(A)). At 24 hpf, *egfl6* expression in the second and third pouches was obvious, with a weak *egfl6* expression appearing in the first pouch (Figure 1(B)). In addition, new *egfl6* expression was observed apparently in a subpopulation of mesodermal cells between pouches at 24 hpf (arrows in Figure 1(B)). *egfl6* expression in the developing hindbrain continued but was reduced (asterisks in Figure 1(B)). At 30 hpf, *egfl6* expression in the developing pouches continued, whereas the mesodermal expression of *egfl6* between pouches disappeared (Figure 1(C)). In addition, *egfl6* expression in the hindbrain was not seen at 30 hpf (Figure 1(C)). Although *egfl6* expression was weak in the fifth pouch, *egfl6* was expressed in all pouches at 36 hpf (Figure 1(D)), with new *egfl6* expression being seen in unidentified tissues adjacent to the first and second pouches (asterisks in Figure 1(D)). While it was suggested previously that the pharyngeal tissue expressing *egfl6* at 28 hpf was the pharyngeal arches (Wang et al. 2015), our analysis of *egfl6* expression in conjunction with a pharyngeal endoderm transgenic reporter indicates that *egfl6* is expressed in the pharyngeal pouches from 18 to 36 hpf. Given the importance of pouches in craniofacial development, *egfl6* expression in pouches may be required for craniofacial development through pouch development.

### Generation of loss-of-function mutations in *egfl6*

**Figure 2.**
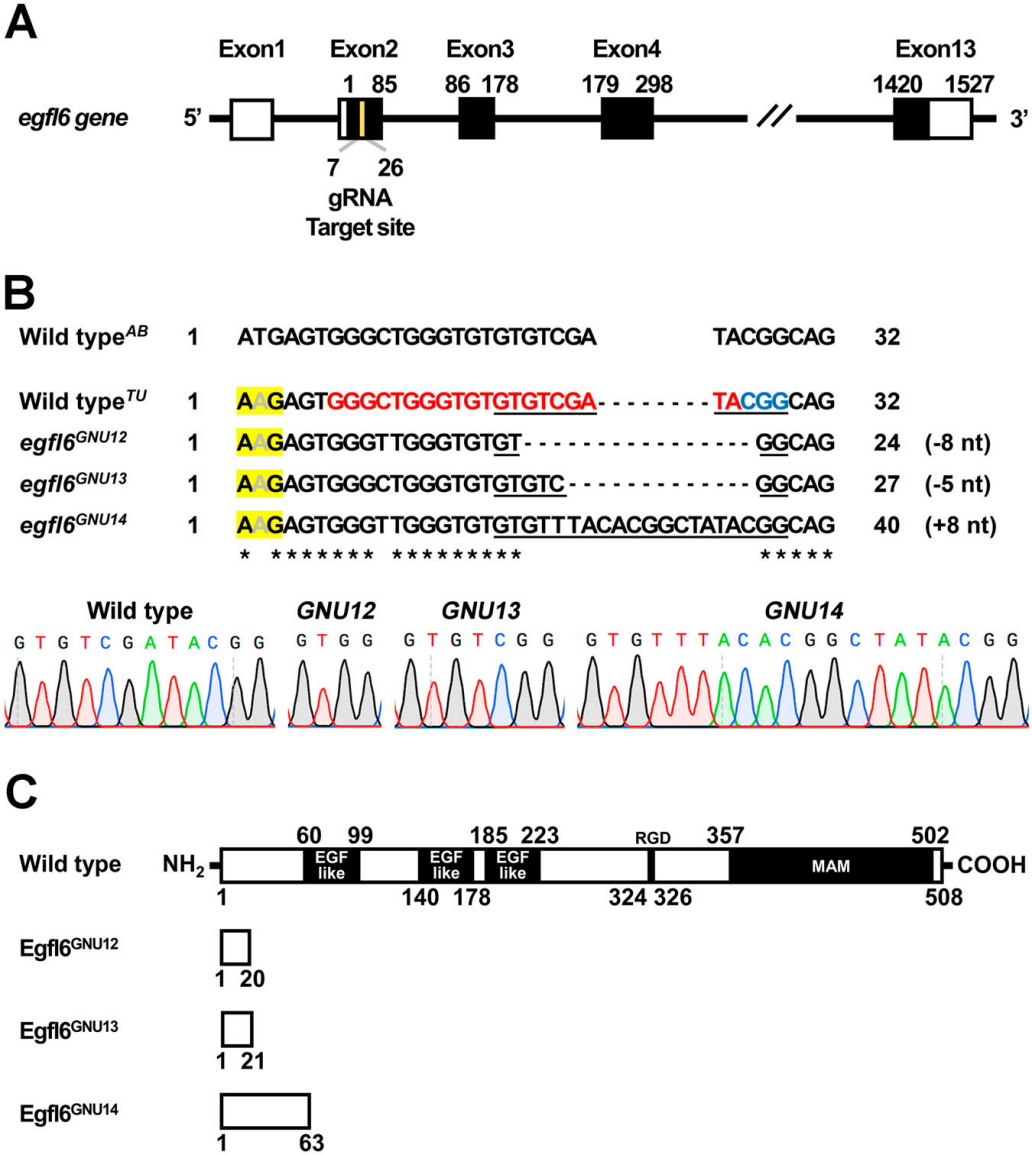
Generation of loss-of-function mutations in *egfl6* gene. (A) Structure of *egfl6* gene. *egfl6* gene consists of 13 exons bearing sequences for the protein-coding region (black box) and the 5′ and 3′ untranslated regions (open box). The gRNA target site in the second exon is marked in yellow. (B) Mutant alleles of *egfl6* gene. The in/del mutation of each mutant allele is shown in the multiple sequence alignments, with the gRNA target and the PAM sites being marked in red and blue, respectively in the wild-type*^TU^ egfl6* sequence. The electrophoretograms show the lesion in each *egfl6* mutant allele that is underlined in the multiple sequence alignments. The start loss mutation in *egfl6* gene in the wild-type TU strain is highlighted in yellow. (C) Schematic of the Egfl6 protein encoded by the wild-type and mutant alleles. The conserved five domains marked in the wild-type Egfl6 protein are missing in all mutant Egfl6 proteins.

To access the function of *egfl6* in craniofacial development, we induced loss-of-function mutations in the *egfl6* gene with CRISPR/Cas9 system. *egfl6* consists of thirteen exons, which encode the conserved five domains, including three EGF_like domains, RGD (Arg-Gly-Asp) domain, and MAM (meprin/A5-protein/PTPmu) domain (Yeung et al. 1999; Wang et al. 2015). A gRNA targeting nucleotides 143–162 from the transcription start site of *egfl6* was designed with ZiFIT (Figure 2(A)). We secured three mutant alleles of *egfl6* (*egfl6^GNU12^*, *egfl6^GNU13^*, *egfl6^GNU14^*) (Figure 2(B)). While wild-type *egfl6* encodes 508 amino acids, *egfl6^GNU12^*, *egfl6^GNU13^*, and *egfl6^GNU14^* are predicted to encode 20, 21, and 63 amino acids, respectively, because of premature stop codon induced by the in/del mutation in each mutant allele (Figure 2(B,C)). Since the conserved five domains of Egfl6 are missing in the three mutant alleles, they are expected to be null alleles (Figure 2(C)).

### Craniofacial development is unaffected by the loss of *egfl6*

**Figure 3.**
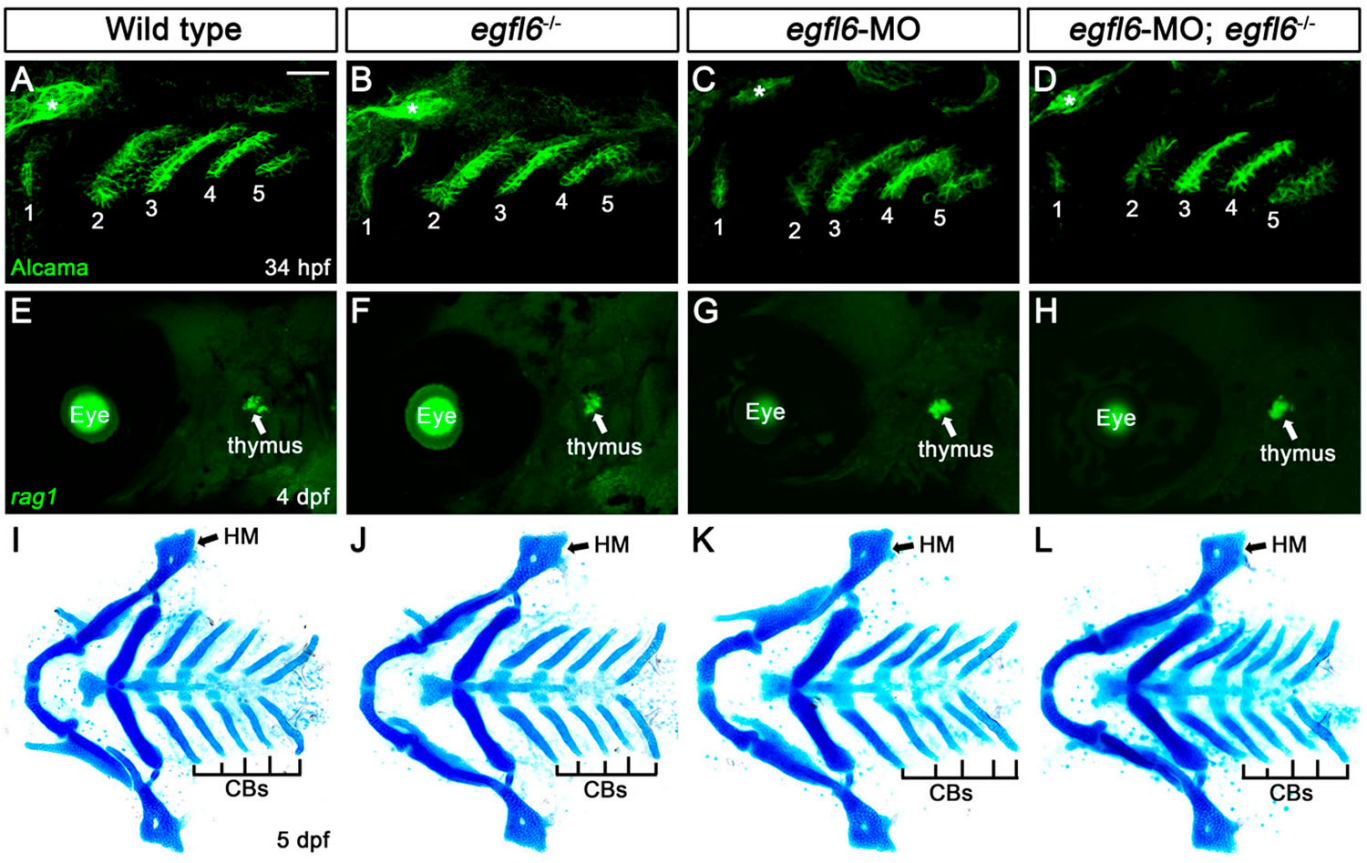
Normal craniofacial development in *egfl6* mutants. (A–D) In both wild-type (A, *n *= 92) and *egfl6* mutant (B, *n *= 31) embryos at 34 hpf, immunohistochemistry for Alcama (green) shows five pouches (1–5). Reduction of *egfl6* with a MO in wild-type (C, *n *= 64) or *egfl6* mutant (D, *n *= 24) animals display normal five pouches. Sensory ganglia are indicated with asterisks. (E-H) Fluorescent *in situ* hybridization for *rag1* (green) at 4 dpf. In both wild-type (C, *n *= 74) and *egfl6* mutant (D, *n *= 29) zebrafish, *rag1* is expressed normally in the thymus. Reduction of *egfl6* in wild-type (G, *n *= 61) or *egfl6* mutant (H, *n *= 17) animals shows normal thymus. (I-L) Ventral whole-mount views of dissected facial cartilages at 5 dpf. Both wild-type (E, *n *= 105) and *egfl6* mutant (F, *n *= 37) zebrafish invariantly form a triangled shape of hyomandibular (HM) and five ceratobranchial (CB) cartilages on each side. *egfl6*-MO-injected animals in wild-type (K, *n *= 88) or *egfl6* mutant (H, *n *= 15) animals have normal facial cartilages, including the HM and CBs. Scale bar: 40 μm.

To investigate the role of Egfl6 in craniofacial development, we first analyzed pouch formation in *egfl6* mutants with Alcama immunohistochemistry. In wild-type animals, five pouches with bilayered structure form at 34 hpf (Figure 3(A)). In *egfl6* mutants, we have not seen any defects in pouch formation in terms of the number and the bilayered structure of pouches at 34 hpf (Figure 3(B)). Since a subpopulation of cells in the third pouch is further differentiated into thymus rudiments (Piotrowski and Nusslein-Volhard 2000), we analyzed whether thymus development was affected by the loss of *egfl6* at 4 dpf. In both wild-type and *egfl6* mutant animals, we have observed the thymus with *in situ* hybridization for *recombination activating 1* (*rag1*), a molecular marker for thymus (Figure 3(E,F)). Normal pouches and thymus seen in *egfl6* mutants suggest that the pouch-specific expression of *egfl6* is not involved in the development of pouches or their derivatives during craniofacial development.

Since pouches are required for the neural crest-derived cells in the pharyngeal arches to differentiate into facial cartilages, including hyomandibular (HM) and ceratobranchial (CB) cartilages (Piotrowski and Nusslein-Volhard 2000; Crump et al. 2004b), we examined the HM and CB cartilages in *egfl6* mutants with Alcian blue staining at 5 dpf. All facial cartilages, including the HM and CB, were normal in wild-type and *egfl6* mutant animals (Figure 3(I,J)), suggesting that *egfl6* expression in the pouches is not required for facial cartilage development. So far, we have not seen any defects in the face of *egfl6* mutants despite the pouch-specific expression of *egfl6* during craniofacial development.

We further analyzed the potential role of *egfl6* in craniofacial development by reducing *egfl6* with an efficient splice-blocking MO (Supplementary Figure 1 and Supplementary Material). Like *egfl6* mutants, reduction of *egfl6* with a MO in wild-type or *egfl6* mutant animals did not affect the development of pouches, thymus, and facial cartilage, confirming that Egfl6 is not essential for craniofacial development (Figure 3(C,D,G,H,K,L)).

### Distinct expression of *egfl7* from that of *egfl6* in pharyngeal region

**Figure 4.**
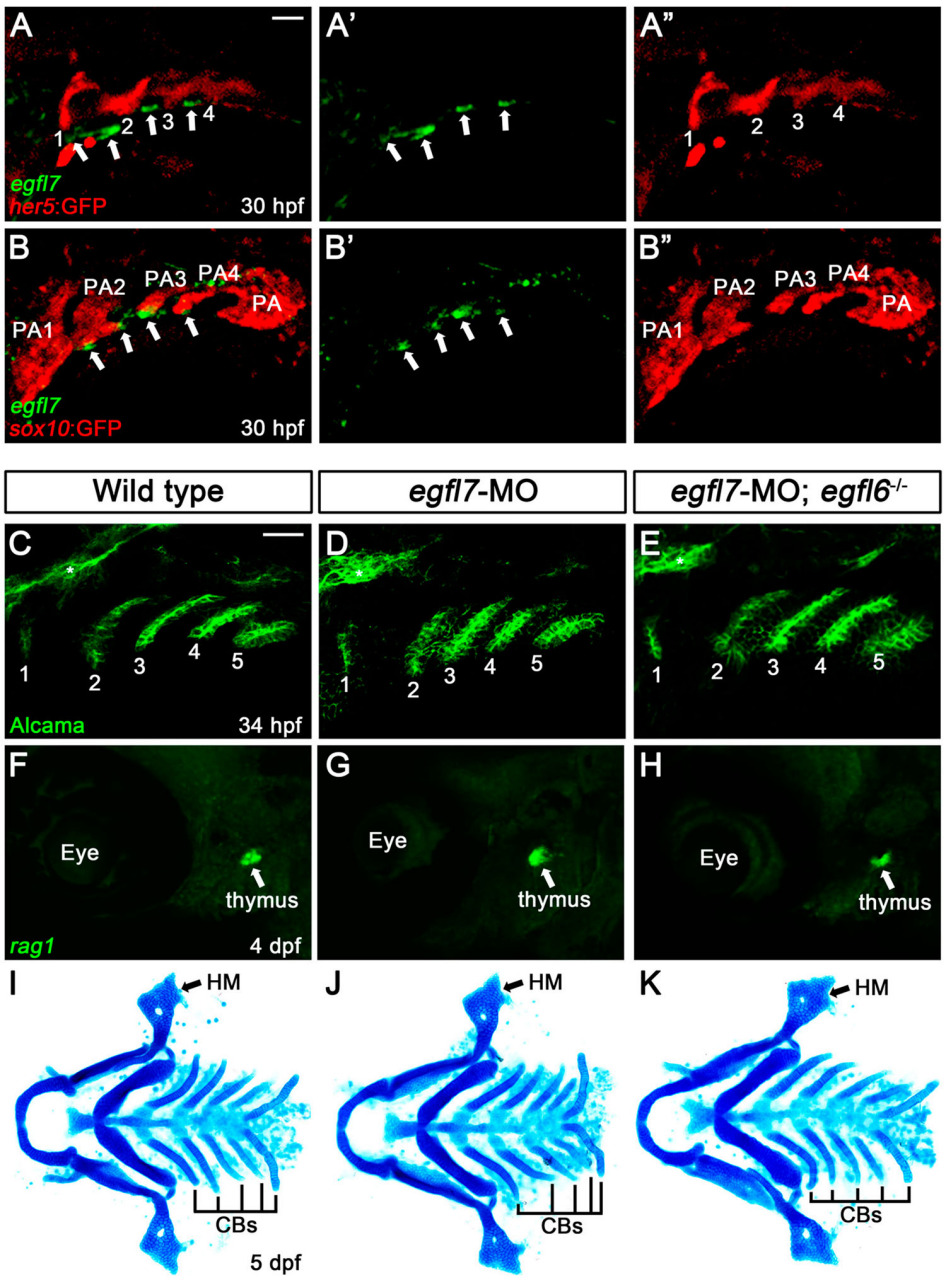
Expression of *egfl7* in the pharyngeal region. (A, B) Fluorescence *in situ* hybridization of *egfl7* (green) in conjunction with the GFP immunohistochemistry (red) in wild-type animals at 30 hpf. (A) *egfl7* is expressed segmentally in small patches (arrows) adjacent to *her5*-positive pouches (1-4). (B) *egfl7* expressing small patches (arrows) are located at the ventral tip of *sox10*-positive pharyngeal arches (PA2-4) but rarely overlapped with PAs. (C-E) Alcama immunohistochemistry (green) labels five pouches (1-5) in wild-type (*n *= 92), *egfl7*-MO (*n *= 80), and *egfl7*-MO-injected *egfl6* mutant (*n *= 21) embryos at 34 hpf. Sensory ganglia are indicated with asterisks. (F-H) At 4 dpf, *rag1* expression (green) in the thymus is normal in wild-type (*n *= 74), *egfl7*-MO (*n *= 76), and *egfl7*-MO-injected *egfl6* mutant (*n *= 14) zebrafish. (I-K) Facial cartilages, including the HM and CBs, are normal in wild-type (*n *= 105), *egfl7*-MO (*n *= 84), and *egfl7*-MO-injected *egfl6* mutant (*n *= 19) animals at 5 dpf. Scale bar: 40 μm.

The normal craniofacial development of *egfl6* mutants could be due to the genetic redundancy with other Egfl proteins. Previously, it has been shown that Egfl6 has similar structural and functional features with Egfl7 (Kang et al. 2020) and that Egfl6 can regulate angiogenesis along with Egfl7 in zebrafish (Wang et al. 2016). To examine potential redundancy of Egfl6 with Egfl7 in craniofacial development, we analyzed the expression of *egfl7* in the pharyngeal region of wild-type *Tg(her5:EGFP)* animal, a reporter of pharyngeal pouch, at 30 hpf; *egfl7* was expressed segmentally in small patches in pharyngeal region, with the small patches rarely overlapped with pouches (arrows in Figure 4(A)). To register the region expressing *egfl7* at 30 hpf, we also analyzed *egfl7* expression in *Tg(sox:EGFP)* reporter that drives GFP expression in the neural crest-derived pharyngeal arches (Carney et al. 2006); the small patches expressing *egfl7* were located at the ventral tip of arches but not in the arches (arrows in Figure 4(B)). Thus, *egfl7* was unlikely expressed in the neural crest-derived arches. Considering the pouch-specific expression of *egfl6* at 30 hpf, *egfl7* expression in the distinct pharyngeal region from that of *egfl6* implies that Egfl6 is unlikely redundant with Egfl7 in craniofacial development. Indeed, reduction of *egfl7* in wild-type or *egfl6* mutant animals with an efficient splice-blocking MO did not affect craniofacial development, including the pouches, thymus, and facial cartilage (Figure 4(C–K), Supplementary Figure 2, and Supplementary Material). Although we still cannot completely rule out a possibility of genetic redundancy of Egfl6 with other Egfl proteins, we suggest that *egfl6* expression in the pouches is dispensable for craniofacial development in zebrafish.

### Endogenous start loss mutation in *egfl6* gene in wild-type Tübingen strain

While we generated loss-of-function mutations in the *egfl6* gene in the background of wild-type Tübingen (TU) strain, we identified an endogenous variant of the *egfl6* gene at the start codon, resulting in start loss mutation in the wild-type TU strain (yellow highlight in Figure 2(B)). The endogenous start loss mutation was unexpected as it was previously reported that the start codon was normal in the wild-type AB strain (Wang et al. 2016). Currently, we could not verify the presence of endogenous Egfl6 protein in the wild-type TU strain due to the absence of antibodies against Egfl6 and the failure of GFP knock-in at the *egfl6* locus. Although we cannot completely rule out a possibility of non-AUG codon usage for normal *egfl6* gene expression, the *egfl6* gene is likely pseudogenized in the wild-type TU strain, further suggesting that *egfl6* is unnecessary for normal craniofacial development.

## Discussion

We have analyzed the expression and function of *egfl6* in craniofacial development. While *egfl6* is expressed in pouches that are key epithelial structures required for normal craniofacial development, loss-of-function mutations in *egfl6* resulted in no defects in the head and face, including the pouches, thymus, and facial cartilages. The normal craniofacial development seen in *egfl6* mutants is unlikely due to the genetic redundancy of Egfl6 with Egfl7 that shares similar structural and functional features with Egfl6. Although we could not determine precisely the region expressing *egfl7* in this study, it is expressed in the non-pouch and non-arch pharyngeal region at 30 hpf. The distinct expression domains of *egfl6* and *egfl7* in the pharyngeal region suggest that the role of Egfl6 and Egfl7 in craniofacial development would be different from each other. Considering together the anatomy of the pharyngeal tissues consisting of the pharyngeal arches and pouches, the ectodermal clefts, and the lateral plate mesoderm (LPM) (Graham 2008) and the well-characterized role of Egfl7 secreted from LPM in trunk angiogenesis (Parker et al. 2004), we speculate that a subpopulation of LPM cells expresses *egfl7* probably for the development of facial blood vessels or facial muscles. Analysis of *egfl7* expression in conjunction with a molecular marker for LPM will determine the pharyngeal tissue expressing *egfl7*.

Previously it was reported that Egfl6 is required for the development of the notochord and blood vessels in the trunk during zebrafish embryogenesis (Wang et al. 2015; Wang et al. 2016). However, our study indicates that the *egfl6* gene carries an endogenous start loss mutation in the wild-type TU strain. Although it is necessary to verify the presence of Egfl6 proteins in the wild-type TU strain with Egfl6 immunohistochemistry or GFP knock-in at the *egfl6* locus, the *egfl6* gene appears to be a pseudogene or non-functional gene at least in the wild-type TU strain. Taken together with the normal craniofacial development of *egfl6* mutants in spite of the pouch-specific expression of *egfl6*, *egfl6* seems to be dispensable for craniofacial development and probably for the normal development in the wild-type TU strain. Since *egfl6* bears the normal AUG start codon in the wild-type AB strain (Wang et al. 2016), comparative analyses of the expression and function of *egfl6* in both the TU and AB strains would provide better insights into the biological roles of *egfl6* in zebrafish development.

## Acknowledgements

We thank Sujeong Gim for technical assistance. This work was supported by the Global Ph. D. Fellowship program through the National Research Foundation of Korea (NRF) funded by the Ministry of Education (2019H1A2A1075288) (to S.J), by Gyeongsang National University academic support (to H. N, H. J, J. P), and by a grant from Basic Science Research Program through the NRF funded by the Ministry of Science and ICT (2019R1A2C1004704) (to C.P.C).

## Supplementary Material

Supplemental Material
