# Supplementary material for: egfl6 expression in the pharyngeal pouch is dispensable for craniofacial development
Source: Anim Cells Syst (Seoul). 2021 Aug 27;25(5):255–63. doi: 10.1080/19768354.2021.1970018 (PMC8567925; doi:10.1080/19768354.2021.1970018)
Supplement: Supplemental Material [file TACS_A_1970018_SM0678.docx]

**Supplementary Figure Legends**

**Supplementary Fig. 1. Validation of *egfl6*-MO.** (A, B) The *egfl6*-MO is designed against the exon 2 (E2)-intron 2-3 splice junction of *egfl6*. In uninjected control embryos, normal splicing of exons 2 and 3 results in excision of intron 2-3 (2624 bp) and loss of PCR amplification of 24-hpf embryonic cDNA by the designated primers (arrows in A). By contrast, 24-hpf embryos injected with 1 nl of 300 μM *egfl6*-MO at the one-cell stage result in amplification of a 500 bp band (*egfl6*-MO PCR), due to a failure to splice out intron 2-3. Red asterisk in (B) marks 500 bp band of a DNA ladder.

**Supplementary Fig. 2. Validation of *egfl7*-MO.** (A, B) The *egfl7*-MO is designed against the exon 3 (E3)-intron 3-4 splice junction of *egfl7*. In uninjected control embryos, normal splicing of intron 3-4 of the *egfl7* gene generates a 519 bp band after PCR amplification with the designated primers (arrows in A). In contrast, one-cell-stage injection of *egfl7*-MO at 300 μM results in a failure to splice out intron 3-4, thus resulting in a 594 bp band (519 bp of exons 2-6 and 75 bp of intron 3-4). Red asterisk in (B) indicates 500 bp band of a DNA ladder.

**Supplementary Materials**

**Primers used to confirm the efficiency of *egfl6*-MO and *egfl7*-MO**

*egfl6*-MO_F: 5’-GCTACGGCTGGAAGAAAA-3’

*egfl6*-MO_R: 5’-TGAGACTTGGCCCAGATA-3’

*egfl7*-MO_F: 5’-ATGTGACCAGCACACCTC-3’

*egfl7*-MO_R: 5’-CAGTGTTCCGTGAGGAAG-3’
